# Which ERP components are effective in measuring cognitive load in multimedia learning? A meta-analysis based on relevant studies

**DOI:** 10.3389/fpsyg.2024.1401005

**Published:** 2024-09-19

**Authors:** Shuyu Yu, Lianghao Tian, Guohua Wang, Shengxin Nie

**Affiliations:** ^1^School of Educational Technology, Northwest Normal University, Lanzhou, China; ^2^School of Educational Technology, Faculty of Education, Henan Normal University, Xinxiang, China

**Keywords:** cognitive load, event-related potentials, multimedia learning, meta-analysis, electroencephalogram

## Abstract

The open and generative nature of multimedia learning environments tends to cause cognitive overload in learners, and cognitive load is difficult for researchers to observe objectively because of its implicit and complex nature. Event-related potentials (ERP), a method of studying potential changes associated with specific events or stimuli by recording the electroencephalogram (EEG), has become an important method of measuring cognitive load in cognitive psychology. Although many studies have relied on ERP output measurements to compare different levels of cognitive load in multimedia learning, the results of the effect of cognitive load on ERP have been inconsistent. In this study, we used a meta-analysis of evidence-based research to quantitatively analyze 17 experimental studies to quantitatively evaluate which ERP component (amplitude) is most sensitive to cognitive load. Forty five effect sizes from 26 studies involving 360 participants were calculated. (1) The results of the studies analyzed in subgroups indicated high level effect sizes for P300 and P200 (2) Analyses of moderating variables for signal acquisition did not find that different methods of signal acquisition had a significant effect on the measurement of cognitive load (3) Analyses of moderating variables for task design found that a task system with feedback was more convenient for the measurement of cognitive load, and that designing for 3 levels of cognitive load was more convenient for the measurement of cognitive load than for 2 levels of cognitive load. (4) Analyses of continuous moderating variables for subject characteristics did not find significant effects of age, gender, or sample size on the results.

## 1 Introduction

Multimedia learning has evolved with the proliferation of educational technology applications and increased opportunities to create multi-channel learning environments (Farkish et al., 2023; Liu et al., 2018; Mayer, 2006). The abundance of verbal and graphic information presented in teaching and learning through various technologies imposes a more complex and challenging cognitive load on learners. Educational researchers must employ advanced designs and techniques to assess cognitive load effectively (Çeken and Taşkın, 2022). Event-related potential (ERP) technology is a measurement technique developed based on EEG, which is able to capture the learners’ EEG activity within a specific time window during the learning process, thus providing a time-series interpretation of the dynamic changes in the cognitive load (Kramer, 1991). However, related studies face certain challenges. Firstly, researchers used different ERP components and measured various brain regions. Secondly, exploration of cognitive load measurement under specific factors and technical conditions has led to variability in findings. Therefore, this study conducted a quantitative review using meta-analysis to integrate existing literature findings, quantitatively assess the validity of ERP components as a measure, and analyze the factors that May affect the experimental results.

## 2 Related work

### 2.1 Cognitive load and ERP technology

Multimedia learning environments offer learners a wealth of information and diverse learning experiences, accompanied by complex cognitive load challenges (Cavanagh and Kiersch, 2023; Mutlu-Bayraktar et al., 2019). Learners often grapple with information overload across various perceptual channels and cognitive dimensions when engaging with multimedia content that incorporates multiple elements such as text, images, sound, and interaction. This complexity necessitates the learner’s cognitive system to not only process increased information but also integrate and interact across different media, thereby intensifying the difficulty of the learning task (Trypke et al., 2023). In response to this challenge, event-related potentials (ERPs) emerge as a particularly advantageous physiological method for directly measuring cognitive load. By recording EEG signals, ERPs can capture a learner’s neuroelectrical activity within specific time windows during the learning process, providing a time-series interpretation of dynamic changes in cognitive load (Ghani et al., 2020). The superiority of ERP in assessing cognitive load within multimedia learning environments is evident in several aspects. Firstly, ERP boasts high temporal resolution, enabling the tracking of rapid brain responses to various stimuli and revealing the temporal characteristics of learners’ processing of multimedia information. It accurately quantifies the distribution and temporal dynamics of cognitive load in learning tasks. Secondly, as a direct physiological measurement, ERP circumvents the limitations of subjective evaluation methods (Anmarkrud et al., 2019). Moreover, ERP proves sensitive to neural responses at varying levels of cognitive load, capturing changes in the brain at the millisecond level (Sun et al., 2022). This positions ERP as an ideal tool for investigating learners’ cognitive load in multimedia learning environments, contributing to a comprehensive understanding of the cognitive challenges in the learning process and providing objective physiological guidance for optimizing multimedia learning design.

### 2.2 ERP components

ERP, in contrast to frequency domain analysis of EEG, involves averaging signals across multiple EEG channels while minimizing or eliminating unwanted EEG activity within a specified time window. The ERP divides components according to their latency after stimulus onset, and is typically named according to their deflection direction and average expected latency (Kramer, 1991). However, the latency of ERP components is influenced by various factors, such as stimulus properties, task requirements, and individual differences. The length of latency does not necessarily directly reflect the cognitive significance of the component. Consequently, in addition to components identified within specific time windows (e.g., P3a, N2b), many ERP components are named based on their functions and features. Examples include RON (Readiness Potential), emphasizing its relevance to action preparation and execution (Berti and Schröger, 2003), MMN (Mismatch Negativity), highlighting its sensitivity to discrepancies between expected and actual stimuli (Kramer et al., 1995), and LPP (Late Positive Potential), denotes its positive potential characteristics and the post-stimulus phase in which it appears (Deeny et al., 2014). Although these metrics have been demonstrated to characterize different memory processing processes, their validity as measures of cognitive load is subject to debate. Therefore, it is crucial to quantify the effects of cognitive load on various ERP components and rationalize the system of ERP components as indicators of cognitive load as a whole.

Rolke et al. (2016) used EEG to measure the amplitude of ERP components such as the P300 while learners were performing a visual search task and showed that the amplitude of these components increased with increasing attentional engagement. Xu et al. (2020) analyzed the EEG of 10 participants during a task using a mental workload estimator developed based on EEG and ERPs, and concluded that the amplitudes of N100, P3a, and RON Decreased significantly with increasing MWL in the n-back condition. Aksoy et al. (2021) combined a virtual reality head-mounted display system with an electroencephalogram to measure ERP components such as P100 and P300, which were shown to be effective in describing the learner’s level of cognitive load when performing tasks using VR. In summary, ERPs are effective in measuring cognitive load in different multimedia learning environments. But at the same time, researchers have used many different ERP components, but which ones are most effective for measuring cognitive load in multimedia learning? This question needs to be addressed urgently.

### 2.3 Potential factors influencing the cognitive load of ERP measures

Recent studies employing ERPs for cognitive load assessment have highlighted various factors influencing the outcomes of measurements in multimedia learning environments. Given the diverse findings, further exploration is essential to identify the moderating factors affecting the validity of ERP measures for cognitive load. Beyond the primary focus on ERP components in this study, it was anticipated that numerous variables in the included studies could impact the final measurements. These variables fall into three categories: signal acquisition characteristics of the study, task design characteristics, and participants characteristics.

#### 2.3.1 Signal acquisition

Based on our literature review, we analyzed three features of signal acquisition that May influence cognitive load measurements: brain region, electrode position and number of electrodes.

Deeny et al. (2014) noted that ERP components from different brain regions contribute differently to measures of cognitive load, suggesting that there May be potential moderating effects of signal acquisition in different brain regions. Identifying regional differences is therefore important for accurate assessment of cognitive load.

Many studies have exclusively measured ERP components using data from midline electrode sites like Fz, FCz, Cz, Pz, etc. These middle line positions, as suggested by Eschmann et al. (2018) and Meltzer et al. (2007, 2008), May offer more interpretative insights into cognitive processes compared to others. We therefore thought it necessary to explore whether the use of midline electrodes was more helpful than other electrodes in measuring cognitive load.

In addition, some studies have achieved unexpectedly positive experimental results by relying solely on ERP data from a single electrode or channel to interpret cognitive load (Sun et al., 2022). We therefore expected to compare the simple method of collecting data using a single electrode with the conventional method using multiple electrodes.

#### 2.3.2 Task design

Various studies have employed diverse task designs to elicit cognitive load. We investigated the impact of three task design variables on ERP measures of cognitive load: number of tasks, number of cognitive load levels, interactive of task systems, form of learning resources.

The distinction between single-task and multi-task designs plays a crucial role. While multi-task designs mimic cognitive load in daily life, offering a comprehensive understanding of brain activity during the processing of multiple tasks, single-task designs enable a clearer analysis of neural activity for specific cognitive tasks (Brunken et al., 2003; Pashler, 1993; Schumacher et al., 2001). Both have their advantages, so we analyzed the number of tasks as a moderating variable to see whether cognitive load induced by multi-tasking is easier to measure than that induced by single-tasking in multimedia learning.

Many studies have designed controlled experiments with more than two groups to measure cognitive load (Daffner et al., 2011; Deeny et al., 2014; Folstein and Van Petten, 2011). We expect to find out whether multiple group experiments are more favorable for measurement. Therefore, in order to determine the necessity of a task with multiple load levels, we analyzed the number of load levels as a moderating variable.

Research has shown that increased learner interaction with the learning system can be effective in managing cognitive load (Darejeh et al., 2022; Paas et al., 2004). The presence or absence of interactivity in task systems is another critical factor. The absence of feedback May heighten cognitive load due to self-doubt or lead to cognitive idleness, as learners lack cues for the next step in the task, influencing experimental results (Clark, 1994). We therefore conducted a moderation analysis by considering whether the task system used by participants was interactive or non-interactive.

Imhof et al. (2011) found that sequential pictures and dynamic videos stimulate learners’ cognition differently and are likely to affect valid measures of cognitive load. Therefore, it is necessary to determine which of the two forms of learning materials is more appropriate for measuring cognitive load.

#### 2.3.3 Participants characteristics

In addition, we analyzed the moderation of gender and age of the participants in the study, as Friedman (2003) and Güntekin and Başar (2007) have shown that gender and age have an effect on EEG. Finally, we also analyzed the effect of sample size on the results of cognitive load measurements, which could avoid small sample effects that could be detrimental to our study.

In this quantitative review of studies, we comprehensively review empirical studies from different databases on the use of ERPs to measure cognitive load in multimedia learning, investigating the ERP components used in these studies as well as other variables involved in the experimental treatment. The following research questions were developed accordingly:

RQ1 Which ERP components are more effective in measuring cognitive load?

RQ2.1 How do characteristics of signal acquisition affect measures of cognitive load?

RQ2.2 How do characteristics of task design affect measures of cognitive load?

RQ2.3 How do participant characteristics affect measures of cognitive load?

RQ3 Did publication bias influence our findings?

## 3 Materials and methods

### 3.1 Literature screening and inclusion

In order to ensure the quality and quantity of the original literature, authoritative English databases such as Web of Science, EBSCO, Science Direct, and Google Scholar were selected for the literature search. The combined logical search statement is TS = (workload or cognitive workload or working memory or mental workload) AND TS = (ERP or ERPs or event related potentials) AND TS = (learning or learner or student) Duplicate documents are removed after the search is completed.

After removing duplicates, studies were screened by title and abstract to exclude those that did not meet the inclusion criteria. When the abstract did not provide enough information, the study had to be screened in full text. Finally, articles with ineligible data were excluded according to the screening criteria, and studies that met all inclusion criteria were included in the meta-analysis.

### 3.2 Data collection

We extracted the following information from each study and coded it using Microsoft Excel: sample size, age, gender, ERP components, electrode position (Is it all on the midline), brain regions, number of tasks, number of levels of cognitive load, interaction, type of learning material.

### 3.3 Calculation of effect size

We conducted our analyses using the Comprehensive Meta-Analysis (CMA) software version 3.0, which is specifically designed for conducting meta-analyses. Following Rosenthal and DiMatteo (2001)’s recommendation, we selected the correlation coefficient (r) as the effect size. CMA software automatically transforms the effect size r into Fisher’s Z, bringing the distribution of r closer to a normal distribution and extending the range of values over the entire real number axis. Consequently, our study adopted the correlation coefficient, r, as the effect size for quantifying the impact of each ERP measure on cognitive load. According to Cohen (2016)’s criteria, the effect size r between 0.10 and 0.30 is defined as a small effect, between 0.30 and 0.50 as a medium effect, and above 0.50 as a large effect.

### 3.4 Heterogeneity test and elimination

Heterogeneity was quantified as the percentage of variation in effect size (i.e., the I^2^ statistic), with values of 75% and above indicating high heterogeneity, 50 to 75% indicating moderate heterogeneity, and 25–50% indicating low heterogeneity (Higgins and Green, 2008). We also assessed this by means of the chi-square test (Cochran’s Q statistic). Considering the diversity of the studies included, a high degree of heterogeneity between studies is expected, plus the sample sizes for each frequency of the study are not sufficiently large, it would be irresponsible to use random effects values to calculate the results for each indicator, with the risk of overestimating the size of the effect (Cooper et al., 2019). Therefore, if the heterogeneity I^2^ was >50% when performing the assessment of each indicator, we would first perform a sensitivity analysis, use the leave-one-out method to exclude studies with excessive heterogeneity, and use a fixed-effects model to derive the final results.

When conducting moderator variable analyses, we analyzed all included studies as a whole, as we wanted to be able to guide specific data collection and experimental design as a whole. Moderator variable analyses in this study examined the effect of a variable (integer or continuous) on the strength or direction of the relationship between cognitive load and measured outcomes (Rosenthal and DiMatteo, 2001).

For publication bias measures we used Egger regression and funnel plots, and if publication bias was shown to exist, we used trim and fill methods (Duval and Tweedie, 2000) to calculate bias-corrected estimates of the mean effect. The number of missing sample sizes was estimated to inform future quantitative studies. Our findings were considered to be more robust if there was no significant publication bias.

## 4 Results

**Figure 1:**
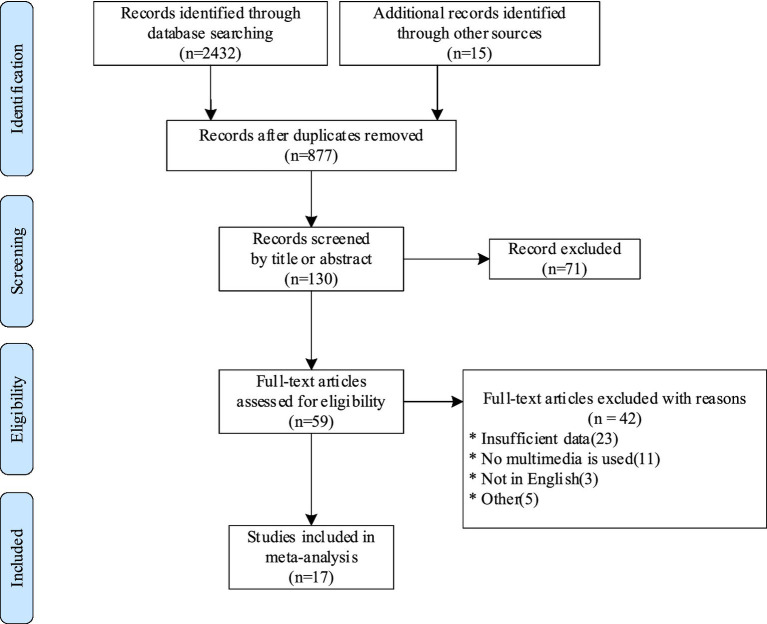
PRISMA study collection flow diagram.

After removing duplicates, 877 records were found in the database and reference list searches. The 130 remaining documents after abstract screening excluded measurements that did not include any ERP component or did not conduct controlled experiments by controlling for different levels of cognitive load. A detailed assessment of the full text of 59 studies identified additional studies that were not included because of the following criteria: insufficient data (23); no use of multimedia technology (11); not in English (3); other reasons (5). In the end, a total of 17 records met the inclusion criteria to be included, see Figure 1 for the process.

### 4.1 Literature screening results

The choice of peak amplitude or mean amplitude in this review depends on which of the two of them better measures differences in levels of cognitive load in Specific studies. In addition to amplitude, four studies were included on latency, which has been widely shown to judge cognitive processing ability (Goodin, 1990; Ford et al., 1980). Sample characteristics, coded moderator variables and effect sizes are shown in Table 1. In addition, because these studies were conducted at the university level, we did not code the stage of the participants, instead coding for age.

**Table 1:** Characteristics of the included literature.

References	Sample size	Age Mean (SD) [range]	Gender	ERPs	Region	Midline M/O	Electros S/M	Task S/M	Load levels	Interaction Y/N	Learning resource	*r*
Aksoy et al. (2021)	21	23.6 (2.1) [20–28]	Mixed (7 F)	N100	Multiple	O	M	S	2	N	Picture	0.010
			Mixed (7 F)	P100	Multiple	O	M	S	2	N	Picture	0.306
			Mixed (7 F)	P300	Multiple	O	M	S	2	N	Picture	0.381
			Mixed (7 F)	P300latency	Multiple	O	M	S	2	N	Picture	0.463
Begum et al. (2014)	30	30.4	–	P300	Parietal	M	S	S	2	N	Picture	0.279
				P300	Parietal	M	S	S	2	N	Picture	0.302
Berti and Schröger (2003)	10	[19–26]	–	P3a	Frontal	M	S	S	2	N	Audio	0.199
				RON	Frontal	M	S	S	2	N	Audio	0.347
				MMN	Frontal	M	S	S	2	N	Audio	0.804
Berti (2008)	12	20 [19–21]	Mixed (10 F)	MMN	Multiple	M	M	S	2	N	Picture	0.303
			Mixed (10 F)	N2b	Multiple	M	M	S	3	N	Picture	0.287
			Mixed (10 F)	P3a	Multiple	M	M	S	3	N	Picture	0.287
Combs and Polich (2006)	16	19.2 (1)	Mixed (8 F)	P3b	Multiple	M	M	S	3	Y	Audio	0.203
Daffner et al. (2011)	37	[18–30] [65–85]	–	N200	Multiple	O	M	S	3	N	Picture	0.363
	18			P300	Multiple	M	M	S	3	N	Picture	0.418
	19			P300	Multiple	M	M	S	3	N	Picture	0.461
	16			P300latency	Multiple	O	M	S	3	N	Picture	0.328
Deeny et al. (2014)	18	26.6 [21–38]	Mixed (11 F)	P200	Frontal	M	S	S	2	Y	Video	−0.482
				P200	Central	Mi	S	S	3	Y	Video	0.657
				P200	Parietal	M	S	S	3	Y	Video	0.478
				P300	Parietal	M	S	S	3	Y	Video	0.558
				LPP	Parietal	M	S	S	3	Y	Video	0.498
Folstein and Van Petten (2011)	16	–	Mixed (6 F)	P300	Multiple	O	M	S	3	N	Picture	0.652
				P300latency	Multiple	O	M	S	3	N	Picture	0.480
				P300	Multiple	O	M	S	3	N	Picture	0.505
				N200	Multiple	O	M	S	2	N	Picture	0.795
Ke et al. (2016)	16	22.63 (1.9) [19–27]	Mixed (7 F)	N200	Multiple	O	M	M	2	N	Picture	0.440
				RON	Multiple	O	M	M	3	N	Picture	0.460
Pratt et al. (2011)	15	[18–30]	Mixed (8 F)	P100	Occipital	O	M	M	3	N	Picture	0.589
				P300	Multiple	O	M	M	3	N	Picture	0.456
Rolke et al. (2016)	20	24.6 (4.4) [19–35]	Mixed (15 F)	N100	Multiple	O	M	S	2	N	Picture	0.702
			Mixed (15 F)	N200	Multiple	O	M	S	2	N	Picture	0.255
			Mixed (15 F)	N2latency	Multiple	O	M	S	2	N	Picture	0.595
			Mixed (15 F)	SPCN	Multiple	O	M	S	2	N	Picture	0.379
			Mixed (15 F)	P300	Multiple	O	M	S	2	N	Picture	0.604
Shaw et al. (2018)	12	28	Mixed (1 F)	P300	Multiple	M	M	M	2	Y	Picture	0.575
Sun et al. (2022)	24	21.5 (1.27) [19–27]	Mixed (13 F)	P200	Frontal	M	M	S	2	Y	Picture	0.546
			Mixed (13 F)	N300	Frontal	Mi	M	S	2	Y	Picture	0.659
			Mixed (13 F)	P300	Parietal	O	M	S	2	Y	Picture	0.855
Suzuki et al. (2005)	12	22.5 (2.5)	Mixed (4 F)	P300	Multiple	M	M	S	2	Y	Video	0.441
Watter et al. (2001)	13	20.7	Mixed (8 F)	P300	Multiple	O	M	S	4	N	Picture	0.626
Wei and Zhou (2020)	25	20.68 (1.91)	Mixed (15 F)	P100	Occipital	O	M	M	2	N	Picture	0.061
			Mixed (15 F)	N200	Frontal	M	S	M	3	N	Picture	0.535
			Mixed (15 F)	P300	Parietal	M	S	M	2	N	Picture	0.403
Xu et al. (2020)	10	[20–26]	Mixed (4 F)	N100	Multiple	M	M	S	2	N	Picture	0.332

Midline M/O = Electrodes on the midline or in other positions; Taks S/M = Single or Multiple tasks; Electros S/M = Single or Multiple Electros; Interactions Y/N = Interactions or Noninterference; r = Pearson correlation coefficient (Effect size).

After the effects are standardized, the combined overall ERP effect size *r* = 0.47 [0.41, 0.53] (*I*^2^ = 27.1%, *Q* = 60.3). A fixed effect size model was used because the heterogeneity was within acceptable limits (*I*^2^ < 50%). It shows that overall ERP components measuring cognitive load have moderate effect sizes.

### 4.2 ERP component

RQ1 Which ERP components are more effective in measuring cognitive load?

**Figure 2:**
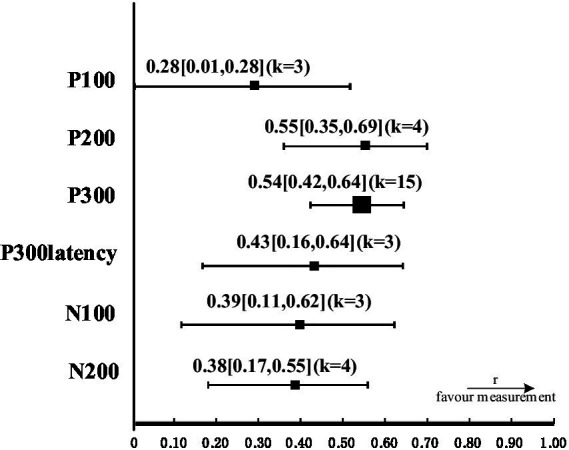
Forest plot of ERP components that effectively measure cognitive load.

The study was analyzed by grouping the studies according to the different ERP components, and the effect size of each ERP component as a measure of cognitive load was calculated, as shown in Table 2. Indicators that lacked sufficient sample size to support them (*k* < 3) could not be judged to be valid, and a forest plot of valid indicators is shown in Figure 2.

**Table 2:** ERP components effect size statistics.

Measurement index			Number of studies	Effect size	Lower limit	Upper limit	*Z*	*p*
Positive wave	P300 component	P300	15	0.54	0.42	0.64	7.62	0.00
		P3a	2	0.25	−0.23	0.63	1.02	0.31
		P3b	1	0.20	−0.33	0.64	0.74	0.46
		P300latency	3	0.43	0.16	0.64	3.05	0.00
	P200		4	0.55	0.35	0.69	4.97	0.00
	P100		3	0.28	0.00	0.51	2.10	0.04
	SPCN		1	0.38	−0.70	0.08	1.65	0.10
	LPP		1	0.50	0.04	0.78	2.12	0.03
Negative wave	N200 component	N200	4 (5–1)	0.38	0.17	0.55	3.50	0.00
		N2b	1	0.29	−0.74	0.34	0.89	0.38
		N200latency	1	0.60	0.21	0.82	2.82	0.01
		MMN	2	0.60	−0.09	0.90	1.74	0.08
	N100		3	0.39	0.11	0.62	2.69	0.01
	N300		1	0.66	0.35	0.84	3.62	0.00
	RON		2	0.42	0.01	0.71	2.01	0.04

#### 4.2.1 P300

Studies that used the P300 to measure cognitive load (*k* = 15) showed high levels of combined effect sizes, *r* = 0.53 [0.32, 0.63], *p* < 0.05, suggesting that P300 amplitudes in the high cognitive load task were significantly lower than P300 amplitudes in the low cognitive load task. Heterogeneity was low, *Q* = 20.41, *p* = 0.118, 𝜏^2^ = 0.28, *I*^2^ = 31.4%.

In subgroup calculations of electrode site effect sizes, r = 0.55 [0.42, 0.66] (*k* = 10) for the multiregional electrode group and *r* = 0.52 [0.21, 0.73] (*k* = 5) for the parietal electrode group, with a non-significant moderating effect (*Q* = 0.30, *p* = 0.843).

#### 4.2.2 P200

The combined effect size for studies measuring P200 (*k* = 4) *r* = 0.55 [0.35, 0.70], *p* < 0.05, suggesting that P200 amplitudes were significantly lower in the high cognitive load task than in the low cognitive load task. Heterogeneity was not significant, *Q* = 0.7, *p* = 0.87, *I*^2^ = 0. Limited by the small number of studies on P200, there were no valid data from subgroup calculations of electrode site effect sizes or precise conclusions to be drawn from related studies.

#### 4.2.3 P300 latency

The combined effect size for studies measuring P300 latencies (*k* = 3) *r* = 0.43 [0.16, 0.64], *p* < 0.05, suggesting that P300 latencies were significantly longer in the high cognitive load task than in the low cognitive load task. Heterogeneity was not significant (*Q* = 0.27, *p* = 0.87, *I*^2^ = 0).

#### 4.2.4 N200

The combined effect size of studies (*k* = 5) using the N200 to measure cognitive load was not credible, *r* = 0.18 [0.02, 0.36], *p* = 0.076 > 0.05. Impact analyses by leave-one-out identified 1 study that was considered an outlier (Wei and Zhou, 2020). Withdrawing it resulted in an increase in mean effect size and a Decrease in heterogeneity, *r* = 0.38 [0.17, 0.55], *Q* = 0.73, *p* > 0.10, 𝜏^2^ = 0.03, *I*^2^ = 0%.

#### 4.2.5 P100 and N100

Few studies have measured cognitive load using the P100 (*k* = 3) and N100 (*k* = 3), with a combined effect size of *r* = 0.28 [0.01, 0.51], *p* = 0.036 for the P100, and r = 0.39 [0.11, 0.62], *p* = 0.007 for the N100. Both showed a Decreasing trend in peak amplitude as cognitive load increased.

LPP, N300, P3a, P3b, RON and SPCN could not be analyzed separately due to insufficient sample size (*k* < 3), but their contributions to the overall ERP effect sizes were still statistically significant.

### 4.3 Moderation variables analysis

#### 4.3.1 Signal acquisition

RQ2.1 How do characteristics of signal acquisition affect measures of cognitive load?

We analyzed the three characteristics of brain regions (*Q* = 3.264, *p* = 0.515), whether there were midline electrodes (*Q* = 0.26, *p* = 0.611) and the number of electrodes (*Q* = 0.08, *p* = 0.775) and found that none of these moderating variables were statistically significant (see Table 3).

**Table 3:** Moderation analysis of signal acquisition as variable.

Moderator variable	Group by	Number of studies	Effect size	Lower limit	Upper limit	*Z*	*p*	*Q*	*p*
Electrodes	S	12	0.456	0.338	0.560	6.86	0.000	0.08	0.775
	M	33	0.427	0.397	0.547	10.44	0.000		
Position	Midline	23	0.455	0.366	0.536	8.95	0.000	0.26	0.611
	Other	22	0.489	0.583	0.495	7.94	0.000		
Regions	Multiple	28	0.445	0.363	0.520	9.56	0.000	3.26	0.515
	Frontal	7	0.552	0.402	0.674	6.22	0.000		
	Parietal	7	0.510	0.293	0.677	4.22	0.000		
	Occipital	2	0.325	−0.256	0.734	1.10	0.269		
	Central	1	0.657	0.274	0.860	3.05	0.002		

#### 4.3.2 Task design

RQ2.2 How do characteristics of task design affect measures of cognitive load?

We analyzed the effect of the characteristics of the task design on the measurements, as shown in the table, the four characteristics of the task system with interactivity (*Q* = 4.840, *p* = 0.028), the number of tasks performed (*Q* = 0.536, *p* = 0.464), the number of levels of cognitive load (*Q* = 8.64, *p* = 0.004), and the type of learning material (*Q* = 2.009, *p* = 0.366). The effect sizes of task system with interactivity, and tertiary cognitive load level were found to have high effect sizes between groups. The effect size of the task system with interactivity (*r* = 0.58) was larger than that of the task system without interactivity (*r* = 0.43); the effect size of the level of tertiary cognitive load (*r* = 0.56) was significantly higher than that of the level of secondary cognitive load (*r* = 0.39), and both of them contributed the vast majority of the between-groups heterogeneity (*Q* = 0.833, *p* = 0.004) (see Table 4).

**Table 4:** Moderation analysis of task design as variable.

Moderator variable	Group by	Number of studies	Effect size	Lower limit	Upper limit	*Z*	*p*	*Q*	*p*
Tasks	Single	8	0.423	0.269	0.555	5.042	0.000	0.54	0.464
	Multiple	37	0.481	0.410	0.546	11.67	0.000		
Load levels	2	25	0.387	0.298	0.469	7.96	0.000	8.64	0.004
	3	19	0.559	0.476	0.632	10.95	0.000		
	4	1	0.626	0.114	0.875	2.32	0.020		
Learning resource	Picture	35	0.459	0.390	0.522	11.59	0.000	2.01	0.366
	Video	6	0.427	0.412	0.711	5.78	0.000		
	Audio	4	0.413	0.033	0.689	2.12	0.034		
Interaction	Interactive	11	0.584	0.462	0.684	7.80	0.000	4.84	0.028
	Noninterference	34	0.427	0.355	0.495	10.43	0.000		

#### 4.3.3 Participant characteristics

RQ2.3 How do participant characteristics affect measures of cognitive load?

As the included ERP studies were all mixed sex and could not be grouped, Meta-regression analyses were attempted with female/all ratio as a covariate, and the results were not significant (*p* = 0.579, *R*^2^ = 0); nor were the results of meta-regression analyses of sample size (*p* = 0.313, *R*^2^ = 0.03) mean age (*p* = 0.929, *R*^2^ = 0) as a covariate, as shown in Table 5. In the analyses of sex ratio as a continuous moderator variable, three studies without a sex description were removed (Begum et al., 2014; Berti and Schröger, 2003; Daffner et al., 2011); in the analyses of age as a continuous moderator variable, the median of the age range was used for the studies that lacked a description of the mean age, which Daffner et al. (2011)‘s study was removed due to the large age span.

**Table 5:** Meta-regression analysis of participants’ characteristics as covariates.

Moderator variable	Covariate	Coefficient	Standard error	95% lower	95% upper	*Z*	2-sided *p*
Gender	Intercept	0.464	0.151	0.168	0.759	3.07	0.002
	Female/ALL	0.147	0.265	0.372	0.666	0.55	0.579
	*R*^2^ = 0	*Q* = 0.31					
Sample size	Intercept	0.640	0.134	0.377	0.9037	4.76	0.000
	Sample size	0.001	0.006	−0.019	0.006	−1.01	0.313
	*R*^2^ = 0	*Q* = 1.02					
Age	Intercept	0.448	0.384	−0.306	1.201	1.16	0.244
	Average age	0.001	0.016	−0.031	0.033	0.06	0.953
	*R*^2^ = 0	*Q* = 0					

### 4.4 Publication bias

RQ3 Did publication bias influence our findings?

**Figure 3:**
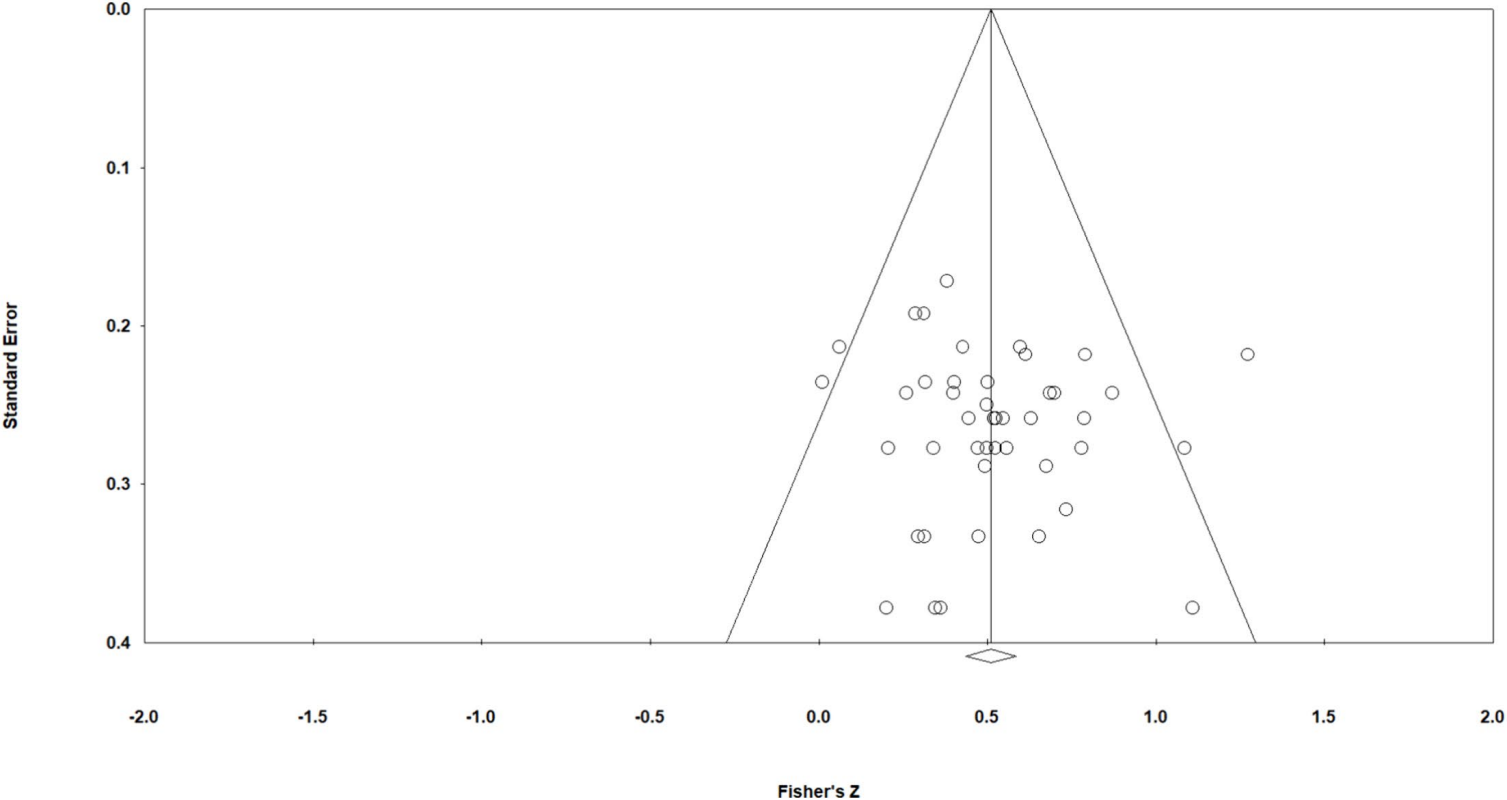
Funnel plot for testing publication bias.

The funnel plot formed by the effect sizes of the ERP primary literature selected for this study was evenly distributed on the left and right with *Z* = 0.51 as the axis of symmetry, indicating that the selected primary literature publication bias was acceptable, as shown in Figure 3. Meanwhile, the test results of Egger’s regression method showed that *t* = 0.60, *p* = 0.55 > 0.05, indicating that the selected original literature publication bias is not significant, so the results of this study are robust.

## 5 Discussion

In our study, we summarize articles that have investigated ERP components as well as event-related potential metrics under different cognitive loads. Firstly, our meta-analysis quantified the overall effect of cognitive load on the ERP components, followed by group analyses of the P100, P200, P300, P300Latency, N100, and N200 components, respectively. A number of moderating variables were found that affected the magnitude of the effect size. The remaining indicators that lacked sample size support and insignificant moderating variables are not discussed further.

### 5.1 ERP components

#### 5.1.1 P300

P300 component is the most used ERP component and is widely used to assess cognitive load in single-task paradigms as well as multi-task paradigms (Kestens et al., 2023). The studies included in this meta-analysis that used the P300 amplitude to measure cognitive load all reported that it Decreased as cognitive load increased, and a high level of effect sizes (*r* = 0.54 > 0.50), coupled with the fact that it is well grounded in sufficient research (*k* = 15) to be judged as the preferred ERP indicator for measuring cognitive load.

#### 5.1.2 P200

P200 component is a positive component of the ERP at the frontal-central scalp position (Zuckerman et al., 2023). Studies have looked at the amplitude of the P200 and found that it consistently Decreases with increasing cognitive workload (Deeny et al., 2014; Sun et al., 2022). It also has a high level of effect size in our results (*r* = 0.55 > 0.50), so it can also be used as a preferred measure of cognitive load.

#### 5.1.3 Other ERP components

Almost all other ERP components had moderate effect sizes in the analyses (0.30 < *r* < 0.50), and P100 had low effect sizes (0.10 < *r* < 0.30). However, they can still be used as alternative indicators to measure cognitive load in multimedia learning environments, serving to complement the experimental data in several ways.

P300 peak latency measures the speed of information processing therefore the latency of the P300 component also shows a direct relationship with cognitive load (Sergeant et al., 1987). The three studies included in this review all found that as cognitive load increased, P300 peak latency also increased (Aksoy et al., 2021; Daffner et al., 2011; Folstein and Van Petten, 2011). To some extent, this suggests that P300 latency can be a good measure of cognitive load.

N200 component is the negative ERP component at the central parietal position of the scalp (Sur and Sinha, 2009). Goodin (1990) were the first to report an increase in the latency of the N200 with increasing cognitive workload. However, in four studies, Ke et al. (2016) found that N200 amplitude Decreased with increasing difficulty, suggesting that N200 is not a stable indicator of performance. Additionally, the research on its peak latency N200latency is very understudied (*k* < 3).

N100 component is a short latency ERP component that is produced primarily by the frontal cortex (Sur and Sinha, 2009). A similar Decrease in N100 amplitude was observed in all three included studies. The results of Kramer et al. (1995) also suggest that N100 amplitude Decreases with increasing cognitive workload.

P100 May be influenced by attention-driven, top-down modulation of visual processing (Gazzaley, 2011). In conditions of high cognitive load, P100 amplitude Decreased. Of the three studies we included, Pratt et al. (2011) increased cognitive load by performing a dual task, which was indeed more experimentally significant than the other two studies. To some extent, this suggests a more favorable triggering effect of the P100 in tasks affecting the allocation of attention.

### 5.2 Moderator variable

In the analysis of signal acquisition method characteristics as moderating variables, none of the three moderating variables were found to be statistically significant. This means that in the included experiments, different electrode numbers, electrode locations or electrode areas caused insignificant or offsetting changes in the measured ERP components. This suggests that we can modestly simplify our signal acquisition operations under the constraints of experimental equipment or time, and that we can measure substantial results by acquiring only the brain regions most relevant to the purpose of the experiment. This statement is not absolute, however, and although these three moderating variables did not show significance, this does not preclude them from playing an important role under specific experimental conditions or tasks. Therefore, further research May need to consider other factors or explore the effects of these variables in more detail.

In the analyses where task design features were used as moderating variables larger effect sizes were found for tertiary cognitive load levels than for secondary cognitive load levels. Experiments that set a level three cognitive load can generate more data and can verify the robustness and consistency of the experimental results by comparing multiple sets of data, thus increasing data complexity and reproducibility, and thus calculating a higher effect size. Analyses on whether the task system was interactive or not found higher effect sizes for task systems with interactivity. It has been shown that increasing learner interaction with the learning system can be effective in managing cognitive load (Darejeh et al., 2022; Paas et al., 2004). Therefore, the design or selection of the task system needs to provide timely feedback to the learners to prevent “cognitive idling” or attentional drift, which May threaten the accuracy of the experimental results.

In the analyses of subject characteristics as continuous moderating variables no significant effects of gender, age, and sample size on the measurement results were found. Preliminarily, it can be concluded that these subjects’ factors do not significantly affect the measurement results in the actual study of multimedia learning.

## 6 Limitations and future work

The lack of adequate sample sizes for some measures to support their effect sizes is likely due to the fact that some researchers only report measures that show statistically significant differences, which prevents the validity of some measures from being confirmed. The lack of sample size is compounded by the fact that there is a lot of specificity in the waveforms within the time window of the ERPs and the tendency for the measurements to be trivial. Therefore, it is necessary to design cognitive load elicitation tests to induce different degrees of cognitive load in subsequent studies, and use them as a basis for extracting and constructing ERP components, or even multimodal measures, in order to effectively characterize learners’ cognitive load.

## Data Availability

The raw data supporting the conclusions of this article will be made available by the authors, without undue reservation.
